# The impact of the rhizobia–legume symbiosis on host root system architecture

**DOI:** 10.1093/jxb/eraa198

**Published:** 2020-04-27

**Authors:** Cristobal Concha, Peter Doerner

**Affiliations:** 1 Institute for Molecular Plant Science, School of Biological Sciences, University of Edinburgh, Edinburgh, UK; 2 Pontificia Universidad Catolica de Chile, Chile

**Keywords:** Legumes, nutrition, rhizobia, roots, root system architecture, symbiosis

## Abstract

Legumes form symbioses with rhizobia to fix N_2_ in root nodules to supplement their nitrogen (N) requirements. Many studies have shown how symbioses affect the shoot, but far less is understood about how they modify root development and root system architecture (RSA). RSA is the distribution of roots in space and over time. RSA reflects host resource allocation into below-ground organs and patterns of host resource foraging underpinning its resource acquisition capacity. Recent studies have revealed a more comprehensive relationship between hosts and symbionts: the latter can affect host resource acquisition for phosphate and iron, and the symbiont’s production of plant growth regulators can enhance host resource flux and abundance. We review the current understanding of the effects of rhizobia–legume symbioses on legume root systems. We focus on resource acquisition and allocation within the host to conceptualize the effect of symbioses on RSA, and highlight opportunities for new directions of research.

## Teaser

Rhizobia improve legume N nutrition, and modulate other aspects of host nutrition and development. Symbiont impact on host resource allocation and root distribution, important for soil resource acquisition, is discussed.

## Introduction Importance of root system architecture for soil resource acquisition

The term ‘root system’ typically refers to the entire root network of a plant, and hence its root system architecture (RSA) is the distribution of this network in space which determines the plant’s capacity to absorb soil resources (de Dorlodot *et al.*, 2007; Tian and Doerner, 2013). Hence, root distribution in the pedosphere is a critical determinant of a plant’s capacity to efficiently, and in competition with other plants, capture below-ground resources. Most resources in soils are heterogeneously distributed and some, such as phosphorus (P) or iron (Fe), are also immobile and therefore roots must be in close proximity to deposits for acquisition (Vance, 2001). Other, more mobile resources, such as nitrate and water, are soluble and percolate into deeper layers. It follows that distinct RSAs are optimal for acquisition of different resources (e.g. deep rooted for water uptake and shallow rooted for uptake of phosphate) (Ho *et al.*, 2005; Uga *et al.*, 2013). Under combined stresses (e.g. low P and drought), a dimorphic root system performs best (Ho *et al.*, 2005). Plants must therefore make architectural trade-offs when roots need to acquire multiple resources with distinct distribution patterns in the soil. This architectural plasticity is underpinned by changes to the growth behaviour of distinct roots and is essential to optimize plant resource acquisition. RSA is therefore not only a reflection of an individual plant’s (external) resource acquisition strategy in its specific environment, but also a history of the (internal) resource allocation (investment) to optimally capture below-ground resources.

**Fig. 1.**
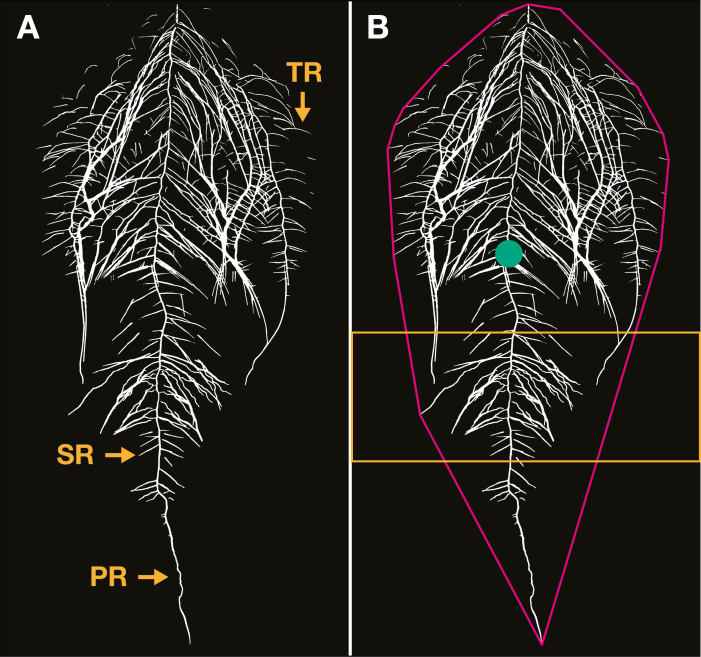
Comparison of traditional and architectural root system analysis. (A) Traditional root system analyses prioritize hierarchical and quantitative relationships between root classes without consideration of their spatial distribution. Thus, the primary root (PR) gives rise to secondary roots (SRs), which in turn give rise to tertiary roots (TRs), and so forth. All roots apart from the PR are collectively termed lateral roots. Measurements usually include number, average, and total length for each root class, and sometimes parameters that refer to the whole root system such as total root length and area. (B) In contrast, architectural analysis does not emphasize root rank but prioritizes their distribution in space to determine their functional contribution to the acquisition of soil resources. Major RSA parameters that describe said shape are the convex hull (the area of the smallest polygon covering the whole root system when projected in 2D, which represents the area the roots are exploring) (magenta); the centroid (the centre of mass of the root system bounded by the convex hull) (green dot); vertical root density (length or area of roots in a given area or volume, which indicates the intensity of soil exploitation in that space) (an example of such a segment is shown between the yellow lines); and other parameters such as total length, area, and depth of the root system. The image corresponds to a chickpea root system captured by the system of Bontpart *et al.* (2019, Preprint).

The characterization of root systems based on their architecture, using parameters which describe and quantify its shape, extent, and density as well as changes over time, which can only be measured *in situ* in the pedosphere, can inform on the plant’s resource capture and use strategies (Fig. 1) (Burridge et al., 2016, 2017; de Dorlodot *et al.*, 2007; Tian and Doerner, 2013). However, such RSA-centric approaches are still not widely employed, probably because many methods used to acquire data, such as X-ray tomography, are costly and inaccessible to many (Kumi, 2015; Metzner *et al.*, 2015). Other methods, which do not consider the positional information of roots in the soil, are more common, since these data are easier to obtain (Fig. 1). For example, most studies analyse the root system in terms of parameters such as length or mass, or as different root classes [e.g. number and length of primary roots (PRs) and lateral roots (LRs)] (Lynch, 2007; Lynch and Wojciechowski, 2015).

## Importance of biotic interactions for root system architecture

Since RSA is directly tied to the capacity of the plant to acquire soil resources, biotic interactions where microorganisms improve plant resource acquisition might have an impact on it. For example, the application of P-solubilizing microorganisms under P-limited conditions might alter RSA due to the reduced need of the plant to exploit heterogeneously distributed P-rich patches. Symbioses between plant roots and bacteria and/or fungi, which entail a direct exchange of nutrients and host-derived resources, for example carbon (C)-rich compounds such as sugars and amino acids, are likely to have a bigger impact on RSA. Therefore, studying how these interactions modify RSA, as well as nutrient acquisition, shoot and root development, plant biomass, and C fluxes will result in a more comprehensive understanding of the different mechanisms and regulatory pathways through which RSA is regulated. In this review, we will focus on the legume–rhizobia symbiosis as a model for how microorganisms modify RSA.

## The legume–rhizobia symbiosis improves N nutrition under limiting conditions

### Legume–rhizobia symbiosis

**Fig. 2.**
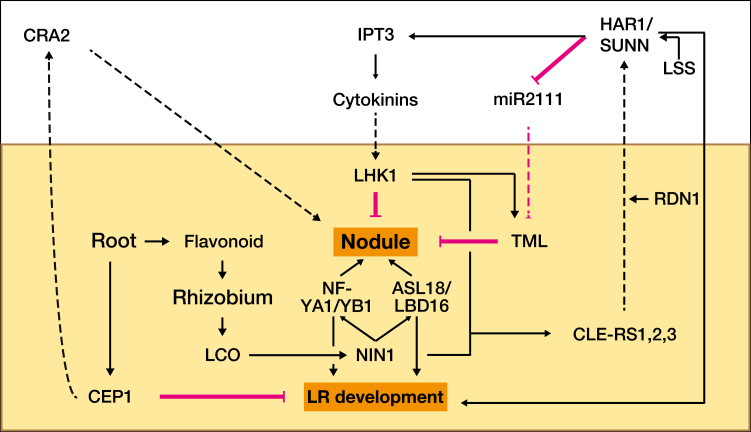
Autoregulation of nodulation (AON). Low N levels induce roots to produce flavonoids which stimulate rhizosphere-associated rhizobia to produce LCO. In *L. japonicus*, these regulate the major transcription factor NIN which activates both NF subunits YA1/YB1 as well as ASL18/LBD16, whose interaction initiates nodulation and nodule development. NIN also induces the expression of genes coding for nodulation-suppressing CLE peptides [mainly CLE ROOT SIGNAL 1 and 2 (CLE-RS1/2)], which are transported to the shoot and perceived by their receptor HAR1. This results in decreased accumulation and shoot–root mobilization of miR2111, which negatively regulates the nodulation suppressor TML. CLE–HAR1 interaction also leads to higher CK synthesis and signalling due to increased expression of IPTG3 and CK translocation to the roots to suppress further nodulation events through their receptor LHK1. This receptor also induces the expression of CLE-RS3, inducing a positive feedback loop on its own regulation, and is required for TML effect in the root cortex. In *M. truncatula*, low N conditions also stimulate roots to synthetize CEP1, which is mobilized to the shoot where its candidate receptor CRA2 positively regulates nodulation. RDN1 arabinosylates rhizobia-induced CLE12, similar to how CLE-RS2 is arabinosylated in *L. japonicus* to interact with HAR1, while LSS (LIKE SUNN SUPERNODULATOR) also further regulates the expression of the HAR1 orthologue SUNN. Mutations in these genes affect LR development since they also participate in the plant response to external N. Finally, NF-YA1/YB1 and ASL18/LBD16 have positive roles in LR development. Together, these genetic interactions show how several of the genes implicated in nodule development and the AON pathway overlap with those that regulate LR development. Black lines with arrowheads signify a positive effect while blunt-ended magenta lines indicate a negative effect. Solid lines indicate regulation while dashed lines signal mobilization to a different plant organ. Nodule and LR development are highlighted and in bold. Light brown indicates below-ground.

Legumes are the second most important group of crops after cereals, accounting for 26% of global crop production, making them an important source of food and income for many (Medeot *et al.*, 2010). Legumes are also a key part of the nitrogen cycle in both agricultural and natural environments since they form symbioses with rhizobia, soil endosymbiotic α- and β-proteobacteria able to fix N_2_ inside modified roots called root nodules (Fig. 2) (Allito *et al.*, 2015; Poole *et al.*, 2018). We use the term rhizobia generically here to include the following genera: *Bradyrhizobium*, *Mesorhizobium*, *Rhizobium*, and *Sinorhizobium*.

When soil N levels, and hence plant N resources, are low, legume roots exude flavonoids that attract compatible rhizobia to roots, where these produce diffusible lipo-chitooligosacharides (LCOs) (Fig. 2). When the plant perceives a compatible LCO, a signalling cascade is triggered that results in the expression of symbiotic genes such as *NODULE INCEPTION PROTEIN (NIN)*. NIN is a transcription factor that increases the expression of both nuclear factor-Y (NF-Y) subunit genes *NF-YA1* and *NF-YB1*, and *ASYMMETRIC LEAVES LIKE 18/LATERAL ORGAN BOUNDARIES DOMAIN 16 (ASL18/LBD16)*. NF-Y and ASL18 proteins interact with each other to relay the rhizobia-mediated signal which ultimately initiates development of the nodule primordia through activation of cell division in the cortex layer (reviewed in Liu *et al.*, 2018; Soyano *et al.*, 2019).

Most rhizobia enter the root through an infection thread, a host-produced structure that guides it from the root hair to the nodule primordium, while others enter by intercellular penetration (crack entry), for example in peanut (*Arachis hypogaea* L.). Endodermis and pericycle cells also form part of the developing nodule; however, these are not infected by rhizobia (Xiao *et al.*, 2014). In the nodule, rhizobia differentiate into a bacteroid and fix atmospheric N_2_ into ammonia, which is protonated into ammonium and captured in organic forms such as glutamine to be used by the plant for growth and development (reviewed in Poole *et al.*, 2018; Ferguson *et al.*, 2019). Nodules can be indeterminate, having a persistent meristem like those of *Medicago* species and pea, or determinate, which do not have an active meristem, such as in *Lotus japonicus* and soybean (Kohlen *et al.*, 2018).

### Similarities between nodule and lateral root development

In model legumes, LRs are predominantly derived from pericycle cells in both indeterminate (Herrbach *et al.*, 2014) and determinate nodule-forming species (Held *et al.*, 2014); however, endodermal and cortical divisions can also be observed (Xiao *et al.*, 2014). In contrast, nodule primordia in *Medicago truncatula* are predominantly founded by the inner cortical cell layers (Xiao *et al.*, 2014). Both organs initiate from their founder cells in response to localized auxin accumulation, and auxin-responsive genes such as the meristem identity genes *WUSCHEL RELATED HOMEOBOX 5* (*WOX5*) and *PLETHORA* are up-regulated at the initiation site of both LRs and nodules. Furthermore, higher expression of auxin response factors and auxin biosynthesis genes such as *YUCCA* are also common to both processes (reviewed in Bishopp and Bennett, 2019). However, while LRs emerge from pre-defined founder cells, nodules are formed in response to LCO perception, which initiates cytokinin (CK)-induced gene expression (reviewed in Schiessl *et al.*, 2019). In *M. truncatula*, CKs, via CYTOKININ RESPONSE 1, promote auxin accumulation in the cortex by increasing the expression of *NIN*, and are also antagonistic to LR development (Schiessl *et al.*, 2019).

Root nodules are modified LRs, and several of the regulatory steps for LR development and nodule organogenesis are shared (Herrbach *et al.*, 2014; Xiao *et al.*, 2014), with 75% overlap in the gene expression changes induced in LRs and nodules (Schiessl *et al.*, 2019). That explains why ectopic expression of *NIN* or mutations in its targets affect both nodule and LR development (Soyano et al., 2013, 2019).

*ASL18/LBD16* has recently been found to be a key link of nodule evolution from LRs (Fig. 2). It is involved in LR development, and in *L. japonicus* it has intronic *NIN*-binding sequences which are conserved in most leguminous *ASL18/LBD16* genes but not in non-leguminous orthologues. These sequences are sufficient for *NIN*-induced *ASL18/LBD16* expression in the nodule primordia (Soyano *et al.*, 2019). Like many LR regulatory genes, *ASL18/LBD16* is induced by auxin, whereas neither *NIN* nor *NF-YA1/2* is. Thus, LRs and nodules share many mechanistic similarities during their early development, indicating that a major part of the LR regulatory programme has been recruited for nodule development during legume evolution. A major unanswered question is how this developmental machinery is harnessed by and responsive to the hosts’ resource acquisition and allocation strategies, and if and how this coupling has changed in the context of nodulation.

### Rhizobia–legume symbioses stimulate growth and enhance plant development

In the presence of compatible rhizobia and in N-limited conditions, entering into symbiosis is usually the most efficient way for legumes to acquire more N. Compared with non-nodulated plants, symbiosis provides a competitive advantage by increasing N levels by up to 5-fold (Solaiman *et al.*, 2011; Wang *et al.*, 2011; Regus *et al.*, 2015; Goh *et al.*, 2016). Rhizobia-dependent N fixation itself is essential for increased plant N levels in N deficit conditions: when two *Lotus* species were inoculated with rhizobial strains with a reduced capacity to fix N_2_, this resulted in a lower increase in plant biomass; rhizobia null mutants unable to fix N_2_ failed to increase plant biomass (Regus *et al.*, 2015; Quides *et al.*, 2017).

**Fig. 3.**
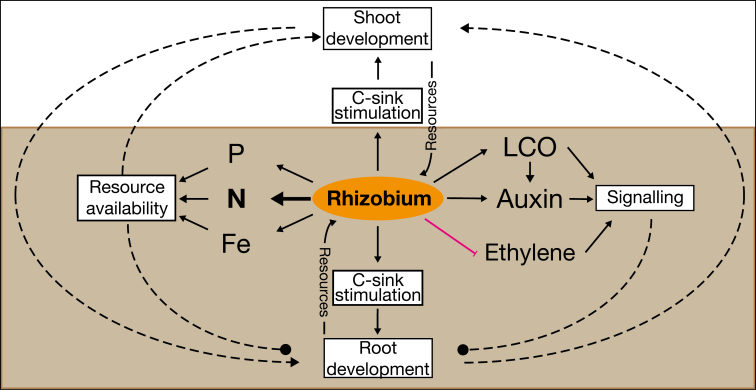
Overview of mechanisms by which rhizobia affects root development. Rhizobia modulate root development through several pathways. The major one is by increasing available N through the fixation of atmospheric N_2_, but it can also increase P and Fe availability by increasing rhizosphere acidity and secreting compounds that mobilize or chelate both compounds, such as siderophores for Fe. In this way, rhizobia contribute to plant resource acquisition, which has a positive effect on shoot development, and its effect on roots will depend on the new nutritional status of the plant to stimulate or inhibit the development of certain roots in certain parts of the root system. Furthermore, rhizobia can synthetize both auxin and LCOs, which further increase root IAA levels, and reduce ethylene concentration by modulating its biosynthesis. The resulting readjustment of the root’s hormonal status alters root signalling which activates/inhibits the initiation and development of specific roots in response to these altered hormone levels. Finally, rhizobia, and the developing nodules, consume C which increases root C sink strength and removes C sink limitation of roots and leaves, thus contributing to increased photosynthesis and enhanced development of both organs. Solid lines indicate a direct positive effect on a specific process, while blunt-ended magenta lines indicate a negative effect. Rhizobia improvement of plant N nutrition is shown in bold to highlight its major contribution to plant development. Text in boxes indicates key processes. Dashed lines with an arrowhead indicate a positive, indirect impact on organ development, while dashed lines with a circle indicate an impact (either positive or negative) on root development. Brown indicates below-ground.

Symbiosis requires the host to allocate C to the symbiont, which in some legumes can comprise up to 14% of their total photosynthates (Kaschuk et al., 2009, 2010*a*), but in low N conditions this allocation is an investment and not a cost: first, under conditions of nutrient, including N, limitation, growth is rapidly uncoupled from photosynthetic C fixation. Declining growth leads to a reduction in sink strength which in turn diminishes the amount of C that growing organs are able to utilize (Fig. 3). Such C sink limitation is caused by the sink organ’s inability to utilize C in adverse environmental conditions (e.g. high temperature or drought) or mineral nutrient deficiencies (e.g. N or P) (Körner, 2015). Secondly, C sink limitation also leads to an accumulation of (unutilized) soluble carbohydrates and starch in source tissues which also negatively impacts photosynthetic C fixation (Paul and Foyer, 2001)

Symbiosis and N_2_ fixation therefore stimulate growth by improving N nutrition and simultaneously reducing or removing the host’s C sink limitation. As a consequence, active symbioses result in higher photosynthesis and C fixation by feed-forward stimulation as reported for soybean (Fig. 3) (Harris *et al.*, 1985; Zhou *et al.*, 2006; Kaschuk *et al.*, 2010*a*), *Lotus* (Regus *et al.*, 2015), and *Vicia faba* (Kucey and Paul, 1982). This probably explains the somewhat paradoxical observation that even in cases where leaf N levels are lower than those of control N-fertilized plants, rhizobia-nodulated plants have higher C fixation. For example, Kaschuk *et al.* (2012) report that symbiosis in soybean increased photosynthesis by up to 31% due to increased sink stimulation and decreased starch and soluble sugar accumulation, which removes carbohydrate inhibition of photosynthesis in source tissues (Azcón-Bieto, 1983; Kaschuk et al., 2009, 2010*a*). Higher yields in nodulated compared with N-fertilized control plants have also been reported in other legumes (Kaschuk *et al.*, 2010b). Increased C consumption in nodules for N_2_ fixation in alfalfa and *M. truncatula* (Larrainzar *et al.*, 2014; Gebril *et al.*, 2015; Kaur *et al.*, 2019), and export of fixed-N metabolites to the host in soybean (Collier and Tegeder, 2012; Carter and Tegeder, 2016) enhanced plant biomass, which stimulated N fixation in turn.

Increased C consumption by nodules should only have a positive effect on photosynthesis when adequate N_2_ levels are fixed to support the higher metabolic demands of the host. Indeed, nodulated common bean, pea, and soybean have higher photosynthetic rates compared with uninoculated plants only at high N_2_ fixation rates (Bethlenfalvay *et al.*, 1978; Zhou *et al.*, 2006; Bambara and Ndakidemi, 2009). Similar findings are reported in pea plants treated with LCOs compared with untreated controls when grown in soil with native rhizobia, which showed a higher photosynthetic rate and N content due to more abundant nodulation and N_2_ fixation activity (Podleśny *et al.*, 2014; Siczek *et al.*, 2014).

Interestingly, nodules that fix higher amounts of N_2_ are also allocated more resources compared with those that fix low quantities, which are penalized by the host in terms of C allocation (Simms *et al.*, 2006; Regus *et al.*, 2015; Westhoek *et al.*, 2017). The most productive symbioses have high sink strength nodules which also allocate high amounts of organic N to the host, thereby creating a strong, positive feedback on plant growth under N-limiting conditions (Kaschuk *et al.*, 2010*a*; Quides *et al.*, 2017). This explains why in many symbioses, nodule biomass is proportional to the N_2_-fixing capacity of the rhizobia (Pampana *et al.*, 2016; Quides *et al.*, 2017).

### N availability *per se* impacts resource allocation to roots

Due to the interdependency of N and photosynthesis, plant N levels are a major factor regulating resource partitioning between shoots and roots in many species (Schortemeyer *et al.*, 1999; Goh *et al.*, 2019). When shoot N availability is limiting, a higher proportion of the plant’s total C will be invested in the root system to underpin foraging and N acquisition to satisfy the shoot’s requirements (Fig. 3). Conversely, when shoot N levels are high, C flux to the roots is proportionally, but not absolutely, decreased. Such modifications to root N allocation are reported in many legumes in response to nodulation such as in several *Medicago* species (Goh *et al.*, 2016), *Acacia melanoxylon* (Schortemeyer *et al.*, 1999), and soybean (Wang *et al.*, 2011), but also in non-legumes such as tobacco (Scheible *et al.*, 1997) and Arabidopsis (Yan *et al.*, 2019) due to higher available N.

Taken together, this is strong evidence that active symbioses can stimulate growth and photosynthesis by multiple mechanisms (Fig. 3): symbioses not only directly enhance host N availability, but also stimulate C fixation via relief from feed-back restrictions on photosynthesis. In rhizobia–legume symbiosis, relief from N and C limitation is co-dependent.

We posit that actively N-fixing symbioses are likely to modify host root systems by modulation of sink strength and resource fluxes, which affects shoot–root resource allocation. Such changes are likely also to affect host plant growth regulator (e.g. auxin) signalling, because enhanced sucrose transport to sink organs via phloem augments the amount of auxin transported by this route (Petrášek and Friml, 2009). A major and largely unanswered question is whether this modification of host root systems is global or, alternatively, leads to changes in its distribution in the pedosphere; that is, changes to RSA.

### Effects of rhizobia symbiosis on legume root system architecture

Active symbioses provide a continuous N supply, decreasing host requirements for N foraging. Consequently, if N is no longer the most limiting resource, host resource partitioning and acquisition are predicted to be correspondingly altered to prioritize the assimilation of other, now more limiting, soil resources such as P. To maximize the host’s return on its overall below-ground investment, it would need to modify its foraging strategy, and hence root development, to efficiently use root-allocated resources to absorb these more limiting resources (reviewed in Kaschuk *et al.*, 2010*a*; Goh *et al.*, 2016; Ferguson *et al.*, 2019). Consistent with this, rhizobial symbiosis increases root depth in common bean (Sofi *et al.*, 2017), which reflects a higher demand for water, and increases growth angle on field-grown soybean, indicating a higher exploitation of the topsoil (Yang *et al.*, 2017), a common RSA response to increase immobile P absorption (Williamson *et al.*, 2001; Péret *et al.*, 2011).

Unfortunately, our current state of understanding of how rhizobia modify legume RSA lacks breadth and detail due to several historical, technical, and conceptual limitations. First, in many experiments, the root system is not studied in detail or not at all (Mishra *et al.*, 2011; Ndakidemi *et al.*, 2011; Quides *et al.*, 2017). Secondly, many experimental growth systems are not explicitly designed to study RSA: for example, root systems in soil-grown plants cultivated in pots or long tubes lose their *in situ* RSA once they are removed for imaging or analysis (Wang *et al.*, 2011; Ravikumar, 2012; Yang *et al.*, 2017). Thirdly, there is a lack of awareness or use of RSA-specific parameters, which describe and quantify the root system within the soil for analyses (Burridge et al., 2016, 2017). Since few data of possible RSA changes in active rhizobia–legume symbioses are available, we will review the effect of symbioses on host nutrition, growth, and nutrient signalling, C acquisition and flux, and root development to then consider their possible impact on root architecture.

## Impacts of rhizobia–legume symbioses on host root development

### Modification of legume root traits by rhizobia

Multiple studies have shown that many rhizobia–legume symbioses modify root traits, irrespective of host or symbiont species (Table 1). The general conclusion from these studies is that rhizobia–legume symbioses positively regulate various aspects of root development. The key question becomes: are these changes to root development more likely to be isometric (i.e. a linear, proportional increase of RSA trait values) or allometric (i.e. changes to scale and relative proportions of RSA parameters)? Although measurements of RSA parameters are generally missing, it is likely that these vary specifically in the course of symbioses. Due to the changing identity of the most limiting nutrient resulting from the provision of fixed N, and the resultant changes to C and N resource allocation, changes to RSA parameters will reflect altered priorities in resource acquisition and therefore are likely to be allometric rather than isometric.

**Table 1.** Effect of different rhizobia on shoot and root system modifications, and N content in different legumes

Host		Symbiont		Growth medium	Shoot trait	Root trait	N changes	Comment	Reference
**Species**	**Varieties**	**Species**	**Strains**						
*Phaseolus vulgaris*	4	*R. leguminosarum*	2	Inert	Weight	Weight	Yes	Higher root weight in 1–2 varieties with 1–2 strains. In one variety both strains increased shoot and root mass	Franzini *et al.* (2010)
	1	*Rhizobium* spp. and *R. tropici*	7	Soil mix	Weight	Weight, length	Yes		Karaca and Uyanöz (2012)
	6	*R. phaseoli*	1	Soil mix	Weight	Weight, depth	NR	Higher weight/depth in 5–6 varieties. Higher shoot and root biomass in 4 varieties	Sofi *et al.* (2017)
	1	*R. phaseoli*	1	Soil	Weight	Weight, length	Yes	No changes in root length	Stajković *et al.* (2011)
*Glycine max*	2	*Bradyrhizobium* spp.	1	Soil	Weight	Weight, length	Yes	Small increases in root traits. Higher shoot and root mass in one variety, lower shoot/root ratio with nodulation	Wang *et al.* (2011)
	>10	*Rhizobium* spp. and *R. tropici*	1	Soil	Weight	Weight, length, area	NR	Field experiment	Yang *et al.* (2017)
	1	*B. japonicum*	1	Hydroponic	No	Length, area	No		Egamberdieva *et al.* (2017)
	1	*Bradyrhizobium* spp.	1	Hydroponic	NR	Length	NR	Inferred higher root length	Li *et al.* (2015)
*Vigna unguiculata*	1	*Rhizobium* spp. and *R. tropici*	1	Soil mix	Weight	Weight, length	NR		Arumugam *et al.* (2010)
*Vigna mungo*	1	*Rhizobium* spp. and *R. tropici*	1	Soil	Height	Length	Yes		Badar and Qureshi (2012)
	1	*R. japonicum*	1	Soil	Height, leaf and branch number	Number	NR		Ravikumar (2012)
*Vigna radiata*	1	*R. japonicum*	1	Soil	Height, leaf and branch number	Number	NR		Ravikumar (2012)
*Cicer ariеtinum*	1	*Rhizobium* spp. and *R. tropici*	10	Soil	Weight	Weight, length	NR	Higher root development with 3 strains in greenhouse, no changes in field with those 3	Khaitov *et al.* (2016)
	1	*M. ciceri*	1	Soil	Weight	Weight, length	Yes		Moradi *et al.* (2013)
	1	*Rhizobium* spp. and *R. tropici*	4	Soil	Weight	Weight, length	Yes	Higher length with 3 strains, weight with 2 strains. Higher shoot weight and root length in 2 strains	Solaiman *et al.* (2011)
*Lens culinaris*	1	*R. leguminosarum*	1	Soil	Weight	Weight, length	Yes		Mishra *et al.* (2011)
*Arachis hypogaea*	1	*Rhizobium* spp. and *R. tropici*	6	Soil	Height	Length	NR	No changes in field	Sharma *et al.* (2011)
*Vicia faba*	1	*Rhizobium* spp. and *R. tropici*	9	Two soils	Weight	Length	NR	Higher root length in 3 strains, and shoot weight in 2 of them in one soil	Argaw (2012)
*Medicago truncatula*	3	*S. meliloti* and *S. medicae*	2	Hydroponic	Weight	Weight, length	Yes	Higher weight/length in 1–2 varieties with 1–2 strains. Higher shoot and root weight in one variety with one strain	Kallala *et al.* (2018)
*Pisum sativum*	>10	*R. leguminosarum* bv. *viciae*	1	Hydroponic /inert	NR	NR	NR	Positive relationship between nodule establishment and root system growth	Bourion *et al.* (2010)
*Lotus japonicus*	1	*M. loti*	3	Inert	Weight	NR	NR	Rhizobia increases shoot mass by 6- and 17-fold in growth chambers and greenhouse, respectively. Changes in root traits inferred	Quides *et al.* 2017
*Lotus strigosus*	1	*Bradyhizobium* spp.	4	Inert	Weight	NR	Yes	3 strains increased shoot mass by 2- to 6-fold, and N by up to 5-fold. Changes in root traits inferred	Regus *et al.* (2015)
*Medicago truncatula,*	1	*S. meliloti*	1	Inert	Weight	Weight, length	Yes	Stimulated root growth is both observed and inferred.	Goh *et al.* (2016)
*Medicago sativa*	1	*S. meliloti*	1	Inert	Weight	Weight	Yes	Stimulated root growth is both observed and inferred.	Goh *et al.* (2016)
*Trifolium subterraneum*	1	*R. leguminosarum* bv. *trifolii*	1	Inert	Weight	Weight, length	Yes	Stimulated root growth is both observed and inferred.	Goh *et al.* (2016)
*Vicia faba*	1	*R. leguminosarum*	1	Field	Height, branch number	Weight, length	Yes	Higher branch number, but decreased height. No changes in root length	Desta *et al.* (2015)

NR, not reported.

### Rhizobia-modified legume root development is conditional on the host and environment

It is important to note that not every study of rhizobia–legume symbiosis revealed modified root traits compared with uninoculated plants. In peanut (*A. hypogaea* L.), treatment with several rhizobial strains failed to increase root length (Singh *et al.*, 2011), while, in other cases, modified root traits were genotype dependent or specific to certain soils (Franzini *et al.*, 2010; Argaw, 2012; Kallala *et al.*, 2018). In an experiment with shallow- and deep-rooted soybean genotypes, rhizobia inoculation increased root dry mass only for the deep-rooted variety under two contrasting P scenarios, but not in the shallow-rooting variety under both P conditions (Wang *et al.*, 2011). Khaitov *et al.* (2016) analysed several rhizobia–chickpea symbioses grown in saline soil in greenhouse conditions: three strains resulted in higher root mass and length but, when tested in the field, no differences in root length were found. These reports exemplify that rhizobia–legume symbioses, while generally positively impacting, for example, root mass, length, and/or area, also depend on the specific symbiosis (plant variety and rhizobial strain) and soil environment.

### Rhizobia-mediated higher plant N levels impact resource partitioning

In most nodulated legumes with active symbioses, shoot biomass is higher resulting from the co-dependent stimulation of photosynthesis and N fixation (Harris *et al.*, 1985; Kaschuk *et al.*, 2012). Numerous examples suggest that the larger shoot stimulates root development to allow for exploitation of more soil resources to satisfy its higher requirements. Changes in root mass, length, and area would thus reflect a more intensive exploration/exploitation of the soil to provide said resources for increased shoot growth enabled by relief from sink limitation and higher C fixation (Table 1).

The impact of symbioses on source–sink relationships and on shoot and root growth is contingent on the symbiosis delivering substantially improved N nutrition to the host (Table 1). Goh *et al.* (2016) report for *Medicago sativa*, *M. truncatula*, and *Trifolium subterraneum* that higher N levels are significantly and positively correlated with higher total root length and total first-order LR length. For nodulated chickpea, only strains that resulted in the largest increase of shoot N levels led to increases in root length and mass (Solaiman *et al.*, 2011). As a corollary, in adequate soil N levels or with rhizobial strains that fix low amounts of N_2_, symbiosis would contribute little or nothing to host nutrition, hence not leading to increases of shoot biomass or to changes to root traits (Franzini *et al.*, 2010; Singh *et al.*, 2011; Argaw, 2012; Kallala *et al.*, 2018).

Interestingly, Goh *et al.* (2016) also report that reductions to C flux to the roots were observed independent of the rhizobium strain’s ability to fix N_2_; possibly explained by changes to host plant growth regulator homeostasis. For the *T. subterraneum*–*Rhizobium leguminosarum* bv. *trifolii* interaction, altered plant growth regulator distribution or levels may explain how symbiosis modified the proportion of root-allocated resources targeted to PR or LR development, respectively (Goh *et al.*, 2016). However, more evidence is needed to be confident that this is a widespread mechanism, but this report suggests that rhizobia can potentially also sculpt source–sink relationships by altering host growth regulator distribution.

### Rhizobia-dependent N activates plant N utilization and signalling pathways

In non-legume species such as rice (*Oryza sativa*) and Arabidopsis, nitrate and ammonium activate N utilization and signalling pathways, for example by expression of nitrate-responsive genes (reviewed in Fukushima and Kusano, 2014; Krapp *et al.*, 2014; Medici and Krouk, 2014). A key observation from molecular studies of nitrate-responsive gene expression is the co-regulation of N-, C-, and hormone-responsive pathways, leading to an overall higher steady-state level of metabolism, which also affects root development (reviewed in Hu *et al.*, 2019; Medici *et al.*, 2019; Hu and Chu, 2020). In rice, glutamine is the signal required for nitrate- and ammonium-dependent activation of cytokinin-dependent shoot growth (Kamada-Nobusada *et al.*, 2013). These observations suggest that N fixation products in legumes, possibly glutamine, probably modify N, C, and growth regulator signalling pathways, leading to changes in root development.

Taken together, active symbioses lead to enhanced levels of plant metabolism and growth by a combination of several mechanisms: N_2_ fixation relieves sink and source tissue-level limits on photosynthesis and growth, resulting in co-stimulation of N and C metabolism. Augmented steady-state metabolism increases shoot and root growth capacity. However, growth stimulation of roots and shoots is unlikely to be uniform and proportional: in most circumstances, other resources (e.g. water, P, or Fe) will rapidly limit growth. Consequently, root foraging for limiting resources is predicted to alter root growth patterns and timing (RSA) by modifying resource partitioning, including by changing plant growth regulator homeostasis (Fig. 3). While much evidence has reported that shoot and root mass and/or length are affected by active symbioses, it remains an open question how active symbioses change RSA.

## Rhizobia broadly benefit host non-N resource acquisition and metabolism

### Rhizobial symbiosis impacts multiple aspects of legume nutrition and development

Successful symbioses stimulate both host and symbiont metabolism and signalling, resulting in elevated demands on metabolic capacity to underpin enhanced growth. Therefore, when focusing on their metabolism and resource allocation, it is useful to consider both organisms together, as an assemblage of organisms or holobiont; for example, N fixation biochemistry requires elevated levels of Fe and P, and the enhanced metabolism enabled by provision of reduced N to the holobiont increases Fe and P requirements. Therefore, it would be selectively advantageous for rhizobia if they did not fix only N, but would also directly contribute to non-N nutrient acquisition. It is not surprising then that there are many examples where rhizobia are involved in non-N nutrient acquisition for the host (Table 2); and that many also produce plant growth regulators, which can contribute to modifying resource allocation (Abril *et al.*, 2007; Vargas *et al.*, 2009; Qin *et al.*, 2011). Taken together, this multitude of mechanisms involved in the legume–rhizobia symbiosis indicate a much greater contribution by rhizobia to host resource acquisition and partitioning than often recognized (Kaschuk *et al.*, 2010*a*; Ndakidemi *et al.*, 2011; Goh *et al.*, 2016). The multiple benefits to the plant by the rhizobia has led to the suggestion that the temporary rhizobia–host symbiosis is evolving towards a novel N-fixing organelle (Coba de la Pena *et al.*, 2017).

**Table 2.** Effect of rhizobia on P and Fe content in different legumes

Host		Symbiont		Growth medium	Higher P	Higher Fe	Comment	Reference
**Species**	**Varieties**	**Species**	**Strains**					
*Phaseolus vulgaris*	1	*R. leguminosarum*	1	Inert	Yes	NR	Higher P content compared with reference strain	Abril *et al.* (2007)
*Phaseolus vulgaris*	4	*R. leguminosarum*	2	Inert	Yes	NR	One rhizobia strain increased P content in one bean variety	Franzini *et al.* (2010)
*Glycine max*	1	*B. elkanii*	1	Inert	Yes	NR	Higher P content under two N scenarios when fed different insoluble forms of P	Qin *et al.* (2011)
*Cicer aritenium*	1	*Rhizobium* spp.	29	Soil	Yes	NR	23 rhizobial strains increased P content. Additional experiments achieved similar results.	Imen *et al.* (2015)
*Cicer aritenium*	1	*M. mediterraneum*, *M. tianshanense*, and *M. ciceri*	4	Inert	Yes	NR	Higher P content with one *Mesorhizobium* strain	Rivas *et al.* (2006)
*Cicer aritenium*	1	*M. mediterraneum*	1	Soil	Yes	NR	Higher P content at two different levels of P	Peix *et al.* (2001)
*Phaseolus vulgaris*	6	*Rhizobium spp.*	47	Inert	Yes	Yes	Many rhizobial strains that increase shoot mass in several bean varieties can solubilize P and produce siderophores	Abbaszadeh-dahaji *et al.* (2012)
*Hedysarum coronarium*	1	*Rhizobium spp.*	1	Soil	Yes	Yes		Soumaya *et al.* (2016)
*Glycine max*	2	*Bradyrhizobium* spp.	1	Soil	Yes	NR	Increase in P content in a P-inefficient variety under several nutritional scenarios	Wang *et al.* (2011)
*Vigna mungo*	1	*Rhizobium* spp. and *R. tropici*	1	Soil	Yes	NR		Badar and Qureshi (2012)
*Phaseolus vulgaris L.*	1	*R. leguminosarum*	1	Soil/field	NR	Yes	Higher Fe levels under both field and glasshouse conditions	Ndakidemi *et al.* (2011)
*Vigna unguiculata*	1	*B. japonicum*	1	Soil/field	NR	Yes	Higher Fe levels under both field and glasshouse conditions	Nyoki and Ndakidemi (2014)
*Cajanus cajan*	1	*Rhizobium* spp. and *Bradyrhizobium* spp.	20	Inert	NR	Yes	Rhizobia capacity to produce siderophore shows a high correlation with plant Fe content	Duhan *et al.* (1998)
*Cajanus cajan*	1	*Rhizobium* spp.	25	Hydroponic	NR	Yes	High correlation between shoot Fe content and rhizobia siderophore production	Duhan (2013)
*Lens culinaris*	1	*R. leguminosarum*	1	Soil	NR	Yes	High correlation between shoot Fe content and rhizobia siderophore production	Mishra *et al.* (2011)
*Phaseolus vulgaris*	1	*R. leguminosarum*	1	Soil/field	Yes	NR	Higher shoot P content under field and glasshouse growth conditions, higher root P under greenhouse growth conditions	Makoi *et al.* (2013)

NR, not reported.

However, while little is understood about how the legume–rhizobia symbiosis affects host roots, even less is understood about how the various distinct contributions of rhizobia to host metabolism, signalling, and nutrient acquisition sculpt host RSA, which remains an important open question.

### Rhizobia can benefit their host by enhancing assimilation of P and Fe

Many rhizobia can acidify the rhizosphere to stimulate P and Fe solubilization, and/or produce high-affinity siderophores for Fe^3+^ (Table 2) (Duhan *et al.*, 1998; reviewed in Qin *et al.*, 2011; Jin *et al.*, 2014). As these nutrients become more accessible to roots, they contribute to improved host growth (Orozco-Mosqueda *et al.*, 2013; Geetha and Joshi, 2013; Imen *et al.*, 2015). Thus, rhizobia with these traits have a greater impact on legume growth than those lacking them (Table 2) (Abril *et al.*, 2007; Franzini *et al.*, 2010). The advantages to the host in such symbioses were multiplied if amendments were provided to the crop (Table 2) (Peix *et al.*, 2001; Qin *et al.*, 2011). The beneficial effects of rhizobia on P and Fe acquisition and assimilation may be particularly pronounced in calcareous soils with high pH (Table 2) (Abbaszadeh-dahaji *et al.*, 2012; Soumaya *et al.*, 2016). In an interesting report, rhizobia increased P content in a soybean variety with a root system inefficient for P uptake (deep rooted) under low P conditions, but no changes were observed in a P-efficient system (shallow rooted) (Wang *et al.*, 2011), showing that effects on RSA can be conditional on host genotype. Hence, specific rhizobial strains can significantly contribute to P nutrition in many species or varieties in different environmental conditions, especially when their root systems are not optimal for P acquisition.

Furthermore, several rhizobial strains, including some that increase P content, can also increase the uptake of Fe in hosts such as common bean, chickpea, and cowpea under different environmental conditions (Table 2) (Peix *et al.*, 2001; Ndakidemi *et al.*, 2011; Nyoki and Ndakidemi, 2014). The capacity to enhance Fe uptake is tightly correlated with the level and type of siderophore produced by the rhizobia (Table 2) (Duhan *et al.*, 1998; Duhan, 2013). The beneficial effect of enhanced Fe uptake mediated by microbes associated with the host is not restricted to legumes: in non-legume species such as *Zea mays* L., *Pseudomonas* strains able to produce siderophores can also increase Fe content and remove signs of chlorosis (Sharma and Johri, 2003; Singh *et al.*, 2017).

N_2_ fixation itself benefits from rhizobia-mediated stimulation of P and Fe uptake; for example, Fe is required in high quantities for the N-fixing enzyme nitrogenase and other symbiotic proteins (reviewed in Burton *et al.*, 1998; O’Hara, 2001). Therefore, under Fe or P deficit, rhizobia that increase Fe and/or P levels fix more N_2_ than strains that do not, as reported for pigeon pea (Duhan *et al.*, 1998; Duhan, 2013) and chickpea (Singh *et al.*, 2014). Nodulation *per se* also increases Fe absorption: both N_2_-fixing and non-fixing rhizobia, as well as their siderophores, stimulate the uptake and transport of Fe to the shoot, with nodulation also enhancing root Fe-reductase activity (Derylo and Skorupska, 1992; reviewed in Jin *et al.*, 2014). Thus, nodulation by some rhizobia strains contributes to plant P and Fe acquisition in a host-dependent manner, and is likely to have a major impact on host growth and metabolic capacity in conditions where these nutrients are limiting.

### Rhizobia-enhanced N metabolism can modify host nutrient signalling pathways

Higher N availability due to a successful symbiosis is likely also to alter host signalling pathways (Fig. 3): in several non-legumes, NO_3_^–^ induces the degradation of the host phosphate- and nitrate-responsive signalling repressor SPX4 (reviewed in Hu *et al.*, 2019; Medici *et al.*, 2019; Hu and Chu, 2020). SPX4 co-ordinates utilization of both macronutrients and plant growth, and hence any imbalance in the host will lead to enhanced acquisition of the limiting nutrient. In legumes, high NO_3_^–^ availability may directly stimulate P acquisition through a similar mechanism to that seen in rice (Kamada-Nobusada *et al.*, 2013). This function of SPX4 could plausibly explain the higher P levels observed in many nodulated legumes (e.g. resulting from increased P solubilization activity by the roots in N-sufficient plants) (Qin *et al.*, 2011). Stimulation of metabolism and growth based on enhanced resource availability provides the basis for modified root system development (Kan *et al.*, 2015), for example to promote root growth in the relatively P-rich topsoil layer, in addition to the effect of rhizobia on soil P solubilization.

Enhanced availability of limiting P and Fe (Table 2) enables elevated host metabolism, photosynthetic activity, and plant growth, as has been reported for alfalfa and *Lotus* for active symbioses (Li *et al.*, 2013; Regus *et al.*, 2015; Quides *et al.*, 2017). However, these contributions by rhizobia that overcome limiting non N-nutrient levels in the host are likely to be limited to symbioses that fix high amounts of N_2_ (Belane *et al.*, 2014).

### Enhanced assimilation of non-N nutrients mediated by rhizobia affects root traits

Higher non-N nutrient absorption during interactions with rhizobia has been related to changes in root traits: in chickpea inoculated with a strain that solubilizes phosphate and produces siderophores, a higher root length was observed, and this effect was more pronounced when supplied with either insoluble or soluble phosphate (Singh *et al.*, 2014). In *Phaseolus vulgaris* grown in low P soils, treatment with several P-solubilizing rhizobia increased root dry weight (Korir *et al.*, 2017). In two alfalfa varieties grown with insoluble Ca_3_PO_4_, rhizobia enhance root length compared with plants treated with a nutrient solution, with or without N and P (Li *et al.*, 2013). In pigeon pea, rhizobia that synthesize high levels of siderophores have increased root weight compared with strains that produce low levels and with non-inoculated plants (Duhan *et al.*, 1998; Duhan, 2013). Finally, inoculation of peanut and pigeon pea with rhizobia expressing siderophore receptor genes in autoclaved and non-autoclaved soil resulted in increased root mass compared with their non-transformed parental lines in both soils (Arif *et al.*, 2012). These findings suggest that higher non-N nutrient absorption due to rhizobia could have a much more important impact on overall host resource acquisition and partitioning than previously considered, particularly since most studies do not report root traits in detail.

In conclusion, many rhizobia increase P and Fe levels in legume species. A positive impact on plant nutrition and growth, and, in some instances, modification of root traits associated with high P and Fe content, have been observed. We propose that these modifications to the root systems will result from either a reduced need to forage for these nutrients or from more intensive exploration/exploitation if they result from higher host resource demands due to increased metabolism, which may lead to a different RSA. For example, if P is limiting, a rhizobium strain with low capacity to solubilize it will result in an increased exploitation of the topsoil, as seen in the non-legume Arabidopsis (Williamson *et al.*, 2001; Péret *et al.*, 2011), while a strain that solubilizes P will not lead to this change in RSA, since it already makes far more P available to the roots.

## Rhizobia effects on plant growth regulator homeostasis modulate root development and resource acquisition

### Rhizobia modulate plant growth regulator homeostasis

The symbiont and the host wrestle over their share of the resources available to the holobiont. As part of their arsenal, rhizobia have also evolved mechanisms to modulate host growth regulator homeostasis and signalling, thereby affecting its growth, development, and resource allocation. The best studied of these mechanisms are auxin and LCO biosynthesis, and ethylene signalling. Auxin is implicated in many aspects of plants, and specifically root development, nodulation and nodule development, N-mediated control of RSA, and nutrient acquisition (Pacios-Bras *et al.*, 2003; Liu *et al.*, 2018; Lagunas *et al.*, 2019; reviewed in Sun *et al.*, 2017). Ethylene negatively affects root development and nodulation (reviewed in Okazaki *et al.*, 2004; Saleem *et al.*, 2007). LCOs modulate auxin levels which can stimulate LR formation in legumes (Pacios-Bras *et al.*, 2003), and in the non-legume *Brachypodium distachyon* (Buendia *et al.*, 2019) (Fig. 1).

### Importance of auxin-synthesizing rhizobia for root development

Many rhizobia produce the common auxin indole-3-acetic acid (IAA); in some cases, >90% of the strains that nodulate single host species produce it (Antoun *et al.*, 1998; Vargas *et al.*, 2009; Abbaszadeh-dahaji *et al.*, 2012). In *Vigna mungo* and *Melilotus alba*, mature nodules have much higher IAA levels and decreased amounts of its catabolic enzymes than bulk roots; it has been suggested that this IAA might be transported to other tissues to modulate their functions and therefore impact C partitioning within the plant (Datta and Basu, 1998; Ghosh and Basu, 2006) (Fig. 1).

Inoculation of several mung bean (*Vigna radiata*) varieties with symbionts that produce high IAA levels *in vitro* increases root length and mass (Anjum *et al.*, 2011). In *M. truncatula* and alfalfa, a high IAA-producing strain increased the length of the PR and resulted in higher LR development compared with the control, which itself positively correlated with increased nodule number (Pii *et al.*, 2007). Inoculation with an IAA-overproducing strain leads to a higher production of LRs and a more developed *M. truncatula* and chickpea root system (Bianco and Defez, 2010; Bianco *et al.*, 2014; Singh *et al.*, 2014). The highest increase in shoot dry mass in nodulated *P. vulgaris* varieties is observed after inoculation with IAA-producing rhizobia; they might also stimulate bulk root development (Abbaszadeh-dahaji *et al.*, 2012). Finally, in soybean, both auxin and nodulation increase the expression of miR167c, which positively regulates both nodulation and LR number and length (Wang *et al.*, 2015).

However, rhizobia may also directly affect host auxin homeostasis: in non-legume species, root colonization (not nodulation) by rhizobia modifies root auxin signalling which also results in changes to RSA. In Arabidopsis, colonization by rhizobia leads to inhibition of PR growth and a 2-fold enhancement in the number of LRs, primarily through altering auxin signalling (Zhao *et al.*, 2017). The same rhizobial strain has also been shown to increase IAA levels in rice roots, but their root system was not further analysed (Biswas *et al.*, 2000).

### Auxin effects on nutrient acquisition

Auxin is strongly associated with control of host metabolism and growth; therefore, it is not surprising that rhizobia-derived IAA is correlated with higher nutrient acquisition in symbioses. A high IAA-producing rhizobial strain, and roots of *M. truncatula* plants nodulated with it, secrete higher amounts of organic acids compared with its low IAA-producing progenitor strain, resulting in higher P solubilization and therefore absorption (Bianco and Defez, 2010). This strain, and other IAA-overproducing strains, also have increased nitrogenase expression (Imperlini *et al.*, 2009; Bianco *et al.*, 2014), which was highly correlated with increased shoot weight (Bianco *et al.*, 2014). In *Vicia hirsuta*, inoculation with a strain that produces high IAA levels in nodules results in a 2-fold increase in N_2_ fixation, probably also due to higher nodule mass (Camerini *et al.*, 2008). Similar findings were reported by Kaneshiro and Kwolek (1985) where inoculation of soybean plants with a high IAA-producing mutant resulted in enhanced N_2_ fixation compared with its parent strain. Finally, auxin also regulates plant responses to Fe deficiency, and microbial auxins enhance its absorption in legumes under low Fe conditions (reviewed in Jin *et al.*, 2014).

### Rhizobia can interfere with host ethylene synthesis and signalling to modulate nodule development

In many legume species, ethylene levels quickly increase in response to compatible LCO detection and repress nodule development; mutations in ethylene signalling pathways result in hyperinfected and hypernodulating plants (reviewed in Buhian and Bensmihen, 2018; Reid *et al.*, 2018).

Rhizobia can decrease ethylene levels through two mechanisms: first, the production of the enzyme 1-aminocyclopropane-1-carboxylate (ACC) deaminase which metabolizes the ethylene precursor ACC (reviewed in Ahemad and Kibret, 2014), and, secondly, the synthesis of rhizobitoxine, which inhibits two enzymes required for ethylene biosynthesis upstream of ACC (Duodu *et al.*, 1999; Yasuta *et al.*, 1999; Yuhashi *et al.*, 2000). Loss-of-function mutations of symbiont ACC deaminase or its decreased expression reduce nodulation, nodule development, and shoot biomass (Ma *et al.*, 2003; Uchiumi *et al.*, 2004). Furthermore, loss-of-function mutants in rhizobitoxine synthesis also have more aborted and fewer mature nodules (Duodu *et al.*, 1999; Yasuta *et al.*, 1999; Yuhashi *et al.*, 2000). The addition of an ACC deaminase gene to rhizobial species that do not have it or have low activity of this enzyme greatly enhances their ACC deaminase activity (Ma *et al.*, 2004; Tittabutr *et al.*, 2008). These strains result in higher nodule number and shoot dry mass in alfalfa, and have improved competitiveness compared with their wild-type progenitors (Ma *et al.*, 2004), as well as nodule number and size and root mass in *Leucaena leucocephala* (Tittabutr *et al.*, 2008).

Thus, the competition between host and symbiont in controlling ethylene homeostasis at an early stage of the interaction is essential for an optimal symbiosis, and therefore for adequate resource allocation (Ma *et al.*, 2003).

The observation of ACC deaminase activity only in differentiated rhizobia and not in free-living ones further supports this notion (Uchiumi *et al.*, 2004). Furthermore, rhizobia that can modulate ethylene production can decrease its levels in roots of older plants when compared with controls (Yuhashi *et al.*, 2000), raising the possibility that this is another mechanism by which rhizobia could modulate the root system. However, there are also reports where the loss of ACC deaminase does not affect nodulation or nodule development (Murset *et al.*, 2012), suggesting that the sensitivity of the symbiosis to ethylene depends on the plant species.

Although regulation of host ethylene synthesis or signalling by rhizobia is essential for an optimal symbiosis, there is only limited evidence of how this can impact root development: only Tittabutr *et al.* (2008) report increased root mass in *L. leucocephala* when inoculated with rhizobia with high ACC deaminase activity. In contrast, most studies that report legume root traits use co-inoculation of rhizobia with other bacteria that can decrease ethylene production. In lentils, co-inoculation with *R. leguminosarum* and any of two ACC deaminase-producing *Pseudomonas* strains increased both root length and biomass in two different nutritional conditions (Iqbal *et al.*, 2012). Also in this species, inoculation with either a putative *Bacillus* or *Pseudomonas* strain with high ACC deaminase activity increased root weight in seedlings (Saini and Khanna, 2013). Co-inoculating common bean with *R. tropici* and with a transformed endophyte, *Serratia grimesii*, expressing high levels of ACC deaminase, increases both root and shoot weight as well as nodule number, when compared with co-inoculation with a wild-type *S. grimesii*, which does not encode an endogenous ACC deaminase (Tavares *et al.*, 2018).

Thus, rhizobia that possess such mechanisms to decrease ethylene synthesis can enhance the strength of the symbiosis, which is likely to stimulate its impact on legume root development and RSA (reviewed in Okazaki *et al.*, 2004; Tittabutr *et al.*, 2008).

### Regulation of auxin and root development by LCOs

Through production of LCOs, rhizobia can enhance localized IAA content that stimulates LR formation in legumes (Olah *et al*., 2005; Herrbach *et al.*, 2017) and non-legumes (Buendia *et al.*, 2019). In soybean, LCO application also results in an increase in total root length and LR formation (Souleimanov *et al.*, 2002). A higher allocation of shoot resources to the roots has been linked in *M. truncatula* to nodule initiation, which depends mostly on LCO detection (Goh *et al.*, 2016). This may also be due to the high sink strength of the nodules, which in *M. truncatula* is higher than that of both leaves and roots (Jeudy *et al.*, 2010). Finally, higher root biomass is reported at several developmental stages in pea after seeds were treated with LCOs, though this may also be due to increased shoot N and photosynthesis from more intense nodulation (Podleśny *et al.*, 2014).

### Rhizobia modulate holobiont metabolism, growth, and resource acquisition

The capacity of rhizobia to modulate host growth signalling pathways and nutrient acquisition by interfering with, for example, ethylene biosynthesis, changing IAA levels, and producing LCO, raises the possibility that a significant fraction of the effects of active symbioses on the host root system may be caused by these mechanisms rather than by N_2_ fixation itself. Such mechanisms would also result in strong reinforcement of the resource-based effects of symbioses due to the stimulation of host metabolism, which itself enhances nutrient acquisition. Thus, rhizobia probably influence plant C partitioning by increasing C allocation to roots through modifications in auxin and ethylene signalling, resulting in a modified RSA.

## Molecular regulators of rhizobia-mediated modified legume RSA

There is significant conservation of resource-cued signalling mechanisms in LRs and nodule formation (Goh *et al.*, 2016; Lagunas *et al.*, 2019; Schiessl *et al.*, 2019): plant growth regulators, metabolites that function both in signalling and metabolism, and mobile peptide signals and their cognate receptors are involved in both processes (Bensmihen, 2015). In this section, we discuss the role of these signalling mechanisms in nodulation and host root development, with a view to highlighting potential targets of host resource partitioning mechanisms.

### Autoregulation of nodulation

In legumes, two shoot-expressed receptors independently and antagonistically regulate nodulation systemically by perceiving mobile signalling peptides produced in the root contingent on soil N availability and infection status (reviewed in Ferguson *et al.*, 2010; Laffont *et al.*, 2019; Nowak *et al.*, 2019). In the autoregulation of nodulation (AON) pathway, SUPER NUMERIC NODULES (SUNN) in *M. truncatula* or its *L. japonicus* orthologue HYPERNODULATION ABERRANT ROOT FORMATION1 (HAR1) inhibit nodulation by detecting CLAVATA3/EMBRYO SURROUNDING REGION (CLE) peptides, while the likely receptor of C-TERMINALLY ENCODED PEPTIDE (CEP) peptides, the leucine-rich repeat receptor-like kinase (LRR-RLK) COMPACT ROOT ARCHITECTURE2 (CRA2), stimulates nodulation. Furthermore, in *L. japonicus*, *HAR1* modulates nodulation by inhibiting shoot–root mobilization of *miR2111* that represses the nodulation suppressor TOO MUCH LOVE (TML), and by enhancing CK synthesis through activation of ISOPENTENYL TRANSFERASE 3 (IPT3) and translocation to roots to suppress further nodulation events through their receptor LOTUS HISTIDINE KINASE1 (LHK1) (Tsikou *et al.*, 2018). *LHK1* also mediates *TML* responses in the root cortex (Miri *et al.*, 2019). These pathways allow the plant to regulate resource investment into nodule production, and hence are also likely targets of resource partitioning mechanisms (Ito *et al.*, 2007; Murray *et al.*, 2017; Goh *et al.*, 2019). A conceptual model that combines the known regulatory pathways of two legume models (*M. truncatula* and *L. japonicus*) is shown in Fig. 2.

### AON components participate in N response and root development

CLE and CEP peptides have additional roles in regulating LR development, with different local and systemic effects depending on local soil N conditions (Fig. 2) (Araya *et al.*, 2016; Sun *et al.*, 2017; Taleski *et al.*, 2018). A functional AON pathway is also required for roots to perceive, take up, and mobilize N as well as for normal root development (Schnabel *et al.*, 2005; Lagunas *et al.*, 2019). Goh *et al.* (2019) show that *M. truncatula* mutants with loss-of-function alleles of genes involved in regulation of nodulation, such as *sunn*, ROOT DETERMINED NODULATION 1 (*rdn1*), and LIKE SUNN SUPERNODULATOR (*lss*), also have altered biomass allocation, LR length, and density, similar to what Schnabel *et al.* (2005) have shown for *sunn* mutants. This is observed independently of nodulation, which exacerbates these differences due to increased resource competition between roots and nodules. Finally, in *L. japonicus*, the *ROOT DETERMINED NODULATION 1* (*RDN1*) orthologue *PLENTY* and *HAR1* regulate PR length, LR number, and development under both nodulated and uninoculated conditions (Yoro *et al.*, 2019). This reveals a role for the CLE- and CEP-dependent pathway and the AON pathways in the control of C allocation in underground organs (roots and nodules) to acquire nutrients, primarily N (Fig. 2).

Some of these genes evolved in legumes from those required for N status-cued growth regulation, which would explain why they were co-opted into processes related to N acquisition (root development and nodulation). A further example is NIN, whose paralogues are *NIN*-like proteins that mediate nitrate responses in many plant species (Konishi and Yanagisawa, 2013; Suzuki *et al.*, 2013). Another example is the CEP Receptor 1, which directly binds the AtCEP1 peptide to regulate N demand signalling, and is the LRR-RLK most closely related to CRA2 in Arabidopsis (Laffont *et al.*, 2019). Finally, in Arabidopsis, CLE/CEP peptides and their receptors and downstream components have been characterized as important for LR development in response to N (reviewed in Sun *et al.*, 2017; Liu *et al.*, 2020), which suggests that in legumes some of these genes might regulate root development in response to nodulation. Thus, these genes participate in both root system development and nodulation, making it likely that rhizobia will impact root development, to some degree, through these mechanisms.

### Rhizobia-mediated higher shoot nitrogen affects hormone translocation to roots

The effects of rhizobia on root development can also be attributed to higher shoot N levels in nodulated plants, leading to increased shoot–root auxin transport which intensifies LR development (van Noorden *et al.*, 2006; Jin *et al.*, 2012). In *M. truncatula,* this transport is essential to balance C allocation between shoot and roots in response to variable N availability: balancing C allocation for shoot and roots maintains growth homeostasis and depends on the AON gene *SUNN* (Jin *et al.*, 2012; Goh *et al.*, 2016). Higher leaf sucrose levels, both from increased photosynthesis and from elevated C sink strength from the nodules, also have the potential to alter RSA by stimulating auxin synthesis and transport to the roots (Sairanen *et al.*, 2012; Liu *et al.*, 2015).

Nitrate-starved shoots transport CKs to the root where they positively regulate LR development to acquire N (Ruffel *et al.*, 2011). Since in many nodulated plants the shoot has higher levels of N than in non-nodulated plants (Regus *et al.*, 2015; Goh *et al.*, 2016), this may results in reduced levels of CK transported to the roots, leading to decreased LR development. Moreover, glutamine relays the nitrate-dependent induction of several genes that increase CK biosynthesis in shoots (Kamada-Nobusada *et al.*, 2013), and also modulates both root growth and nodulation (reviewed in Mohd-Radzman *et al.*, 2013). Thus, high glutamine levels from N_2_ fixation can have an impact on root growth and nodulation.

Finally, nodulation and LR development share an extensive overlap in their organogenesis and regulatory genes, and both processes share an auxin maximum in the developing organ (Schiessl *et al.*, 2019). This further suggests how nodules evolved as modified roots specialized to acquire N, and how nodulation could alter the expression of genes and hormone levels that impact LR development via changes to resource homeostasis.

## Measuring legume RSA in nodulated plants

### A need to better understand changes in RSA and its dynamics due to rhizobia

As has been shown in this review, many studies report that rhizobia affect several aspects of legume root development in a species- and environment-dependent way (Franzini *et al.*, 2010; Solaiman *et al.*, 2011; Wang *et al.*, 2011), but it is not fully understood how the symbiosis affects RSA due to technical and conceptual limitations. For example, symbioses with different rhizobial strains but similar root mass, length, or area may actually have very different RSA since each strain has a different impact on plant nutrition and C availability and partitioning. Furthermore, the lack of information regarding root growth dynamics may result in changes being masked because roots were not analysed over time (Khaitov *et al.*, 2016; Kallala *et al.*, 2018). Alternatively, it could also mean that some symbioses have a small impact on root development, or they do not result in a modified RSA (Wang *et al.*, 2011; Argaw, 2012; Kallala *et al.*, 2018).

The studies discussed in this review point to the involvement of a combination of altered fluxes of metabolites and signalling molecules that are responsible for changes in legume root development, and therefore possibly changes in RSA. To understand these processes with a view to make the host more resource-capture and utilization efficient, it is evident that detailed studies using systems that allow the study of legume RSA over a long period of time are required.

### Systems to study RSA

Several systems are available to study root development without removing the roots from the soil, allowing study of their *in situ* RSA and growth dynamics. Agar plates are useful to study small root systems in controlled conditions (Laffont *et al.*, 2019; Schiessl *et al.*, 2019), while semi-hydroponic systems, where roots grow attached to a material such as cloth oriented vertically, exist of variable dimensions (15–120 cm) and have been used in many species (Chen et al., 2011, 2017; Lagunas *et al.*, 2019). However, they lack the interaction of roots in soil that more faithfully reflects the natural abiotic and biotic environment in which they evolved (Morris *et al.*, 2018).

**Fig. 4.**
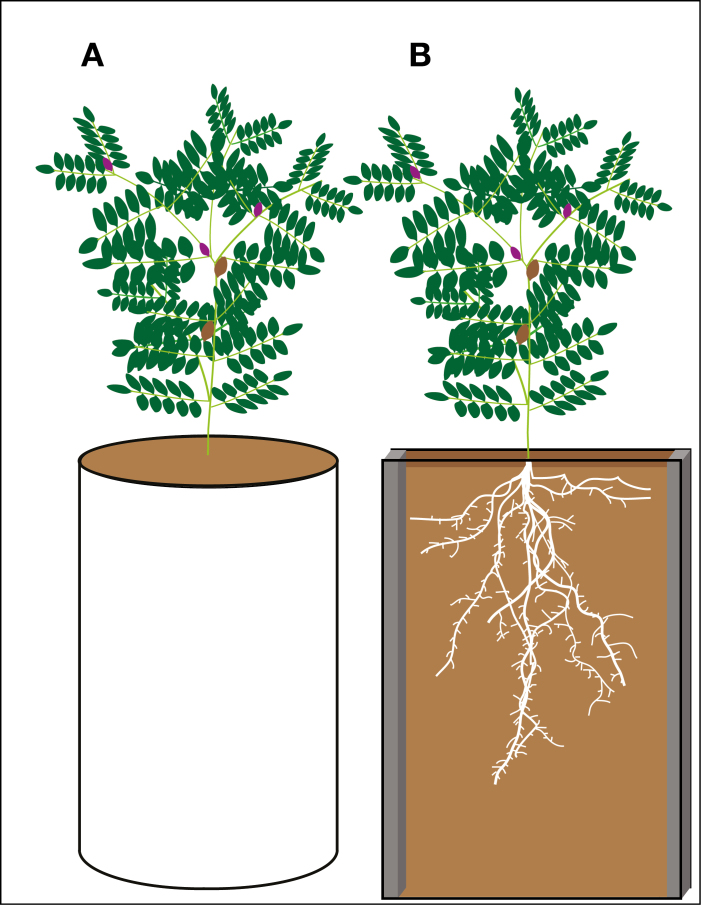
Pot and rhizobox growing systems. (A) Pots and long tubes have been extensively used to analyse roots of rhizobia-treated legumes since they are cheap and easy to use. Depending on size, roots develop as they would in a field, with some horizontal constraints. Roots need to be removed from the soil and washed for imaging, which leads to losses, alters their *in situ* distribution, and precludes repeated measurements on a single plant. (B) Rhizoboxes overcome these limitations and gather high-quality data to continuously analyse root system development and RSA, thereby allowing the study of its dynamics. They have varied dimensions, with at least one transparent side (usually of glass or plastic) that allows capture of root system distribution, either manually or with a camera, without removing them from the growth system. To increase root visibility, rhizoboxes are usually grown at an angle (up to 45°) and their thickness is limited so roots grow in a 2D-like manner. They are also more expensive and have different handling requirements compared with pots and tubes.

The two most common systems used to image roots in soil and overcome its opacity are: (i) X-ray tomography or MRI; and (ii) rhizoboxes. The former systems scan pots up to 80×15 cm and reconstruct a 3D image of the roots, both thick and fine, but are very costly (Kumi, 2015; Metzner *et al.*, 2015). In the latter, roots grow in thin layers of soil (2–40 mm) bordered by a transparent surface so that root development is easily captured with visible wavelength camera(s), and the system is usually inclined up to 45° to maximize visible roots (Nagel *et al.*, 2012) (Fig. 4). However, it can also be placed at 0° if both sides are to be imaged (Rellan-Alvarez *et al.*, 2015). They can be of considerable dimensions (up to 145×45 cm) (Bontpart *et al.*, 2019, Preprint), allowing the study of legumes with large root systems and for long periods of time such as after flowering.

The use of these systems to study RSA in a variety of conditions in many symbioses will provide a better understanding of the role of rhizobia in altering legume RSA. For example, systems of larger dimensions allow for longer periods of unrestricted root growth where more evident changes in RSA could be observed. This would also include those that appear only late in the life cycle, specifically after flowering, when nodulation decreases leaf senescence and enhances C assimilation and allocation to roots (Kaschuk *et al.*, 2010*a*; Li *et al.*, 2016; da Costa Neto *et al.*, 2017). It is not known whether free-living rhizobia in the rhizosphere also contribute to P and Fe acquisition and auxin biosynthesis; it is likely that they do, since they have these effects in gnotobiotic cultures, thus indicating that they are independent of their host (Duhan *et al.*, 1998; Anjum *et al.*, 2011; Qin *et al.*, 2011). Therefore, these systems could determine if and how rhizobia associated with roots contribute to RSA in a significant way.

### Time-lapse study of rhizobia–legume symbiosis RSA

**Fig. 5.**
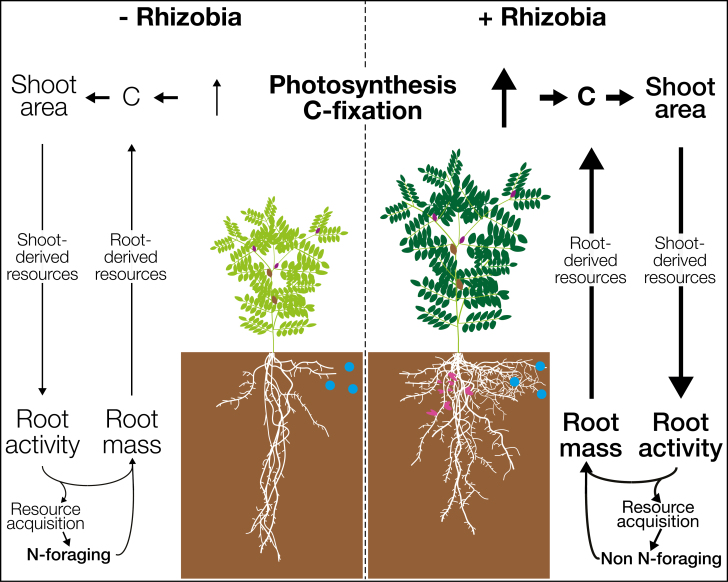
Modification of RSA in legumes due to rhizobia. In low N soil conditions and with no compatible rhizobia (left), legumes need to forage for N themselves; consequently, roots only send relatively low quantities of root-derived resources (specifically N) to the shoot. This leads to low rates of photosynthesis and therefore low levels of C fixation, resulting in slow shoot growth. The shoot will in turn only send low amounts of shoot-derived resources and signals to the roots (e.g. C and auxin along with high levels of CKs). Here, these resources will preferentially be allocated to forage for more N since it limits photosynthesis. When compatible rhizobia are present (right), legumes will enter into symbiosis and produce nodules. These consume C to fix N_2_, leading to higher amounts of root-derived resources transported to the shoot (e.g. N, along with increased levels of P and Fe in some cases). As a result, photosynthesis, and therefore C fixation, will be considerably higher, leading to a larger shoot area. This will result in increased amounts of C and auxin but lower levels of CKs transported to below-ground organs (shoot-derived resources). Hence, the root system will invest proportionally fewer resources to forage for N and more to obtain water and non-N nutrients to satisfy the demand of the larger shoot. As a consequence, changes to root length/area, vertical distribution, and/or exploration/exploitation of different soil layers can be observed in nodulated legumes. Changes in shoot size, and hypothesized changes in RSA, are shown for the nodulated plant. Note how the nodulated plant exploits P deposits more intensively since its N demands (more critical than P demand) are satisfied to a greater extent. Lines and text in bold indicate greater intensity of a specific process. Nodules are indicated in pink, and P deposits are shown as blue circles.

Information regarding how rhizobia modify legume RSA is limited; therefore, we hypothesize here how symbiosis might modify it. Assuming low soil N levels, so the symbiosis is highly beneficial, rhizobia symbiosis will increase plant N levels, photosynthesis, and shoot biomass (Harris *et al.*, 1985; Quides *et al.*, 2017). This will lead to an increase in root mass, length, and area, indicative of higher soil exploitation for resources to satisfy the demands of a larger shoot (Solaiman *et al.*, 2011; Goh *et al.*, 2016). Changes in lateral extension of the root system, and more intense exploitation of the explored soil (root area in the volume/area explored) may be observed due to the higher requirements for immobile resources such as P and reduced need to extensively explore the soil for diffusible N. Depending on water availability, changes in root depth may also be observed, along with higher exploitation of the top layers, a clear indication of a higher P demand. A modified RSA would reveal changes in C partitioning within the root system, indicating where shoot C is being invested underground to forage for resources. A model summarizing how rhizobia modify resource allocation and crosstalk between shoot and roots, and how these changes could affect root development and RSA is shown in Fig. 5.

These modifications will depend on the specific symbiosis, and different strains could result in distinct RSA responses in the same legume variety due to differences in many of their traits (e.g. N_2_ fixation dynamics, P solubilization, IAA production, or C consumption). For example, high auxin-producing strains could increase root C allocation, as well as length and area of the root system compared with strains that produce low IAA levels (Anjum *et al.*, 2011). High IAA-producing strains will also enhance N and P nutrition which would further increase demand for soil resources and therefore root development (Bianco and Defez, 2010; Bianco *et al.*, 2014). Furthermore, such strains could have an impact even when N levels are not low since this mechanism is independent of providing N to the plant. Similarly, strains that strongly acidify the soil would increase P and Fe nutrition (Abril *et al.*, 2007), specifically useful in soil with high pH such as those reported by Soumaya *et al.* (2016). These symbioses should have a lower percentage of roots in top layers than those with strains with limited capacity to acidify the soil since they have less need to intensively exploit the soil for these immobile resources. Thus, they could have a higher lateral extension and depth due to the greater need for resources such as water to support growth of the increased shoot resulting from improved C and N fixation. On the other hand, strains that have high C consumption but fix low N levels, or do not fix at all, may possibly result in root systems with few and long PR and LRs since the plant needs to intensively forage the soil for N, and thus its RSA should be similar to that of non-nodulated plants.

## Summary and conclusions

Legumes are important crops due to their ability to establish symbioses with rhizobia that allow them to fix N_2_. However, little is known about how they modify plant resource partitioning and root foraging strategies, and hence RSA. By positive feedback, highly active symbioses stimulate photo-assimilation due to globally enhanced host metabolic capacity. Modification of root traits (such as weight, length, and area) upon symbiosis depends on both partners as well as environmental conditions. Rhizobial symbioses can enhance not only host N but also macro- and micronutrient availability, specifically P and Fe, and some have the ability to additionally sculpt host growth and resource flux and partitioning by producing plant growth regulators such as auxins. Therefore, effective symbioses result in extensive and complex changes that permit the host to modify resource allocation patterns to roots such that its RSA optimizes acquisition of limiting soil resources. Hence, interactions with other microorganisms, such as mycorrhiza and free-living soil bacteria, might also impact RSA depending on which mechanisms and regulatory pathways are stimulated and to what degree. Therefore, RSA embodies host resource partitioning decision making, as well as rhizobia-modified legume nutrition, C flux, and hormonal signalling. This highlights the great utility of time-series experimental studies of RSA in nodulated and non-nodulated hosts to inform on resource partitioning mechanisms, required to develop more resilient and resource-efficient legume and non-legume crops.

## Abbreviations

ACC: 1-aminocyclopropane-1-carboxylate
AON: autoregulation of nodulation
ASL18/LBD16: ASYMMETRIC LEAVES LIKE 18/LATERAL ORGAN BOUNDARIES DOMAIN 16
CEP: C-TERMINALLY ENCODED PEPTIDE
CK: cytokinin
CLE: CLAVATA3/EMBRYO SURROUNDING REGION
CRA2: COMPACT ROOT ARCHITECTURE2
HAR1: HYPERNODULATION ABERRANT ROOT FORMATION1
IAA: indole-3-acetic acid
IPT3: ISOPENTENYL TRANSFERASE 3
LCO: lipo-chitooligosacharide
LHK1: LOTUS HISTIDINE KINASE1
LR: lateral root
LRR-RLK: leucine-rich repeat receptor-like kinase
NF-Y: nuclear factor-Y
NIN: NODULE INCEPTION PROTEIN
PR: primary root
RDN1: ROOT DETERMINED NODULATION 1
RSA: root system architecture
SUNN: SUPER NUMERIC NODULES
TML: TOO MUCH LOVE
